# Sentence Context Differentially Modulates Contributions of Fundamental Frequency Contours to Word Recognition in Chinese-Speaking Children With and Without Dyslexia

**DOI:** 10.3389/fpsyg.2020.598658

**Published:** 2020-12-03

**Authors:** Linjun Zhang, Yu Li, Hong Zhou, Yang Zhang, Hua Shu

**Affiliations:** ^1^Beijing Advanced Innovation Center for Language Resources and College of Advanced Chinese Training, Beijing Language and Culture University, Beijing, China; ^2^Department of Applied Psychology, Beijing Normal University-Hong Kong Baptist University United International College, Zhuhai, China; ^3^International Cultural Exchange School, Shanghai University of Finance and Economics, Shanghai, China; ^4^Department of Speech-Language-Hearing Sciences, University of Minnesota, Minneapolis, MN, United States; ^5^Center for Neurobehavioral Development, University of Minnesota, Minneapolis, MN, United States; ^6^National Key Laboratory of Cognitive Neuroscience and Learning, Beijing Normal University, Beijing, China

**Keywords:** dyslexia, word recognition, semantic context, *F*_0_ contours, noise

## Abstract

Previous work has shown that children with dyslexia are impaired in speech recognition in adverse listening conditions. Our study further examined how semantic context and fundamental frequency (*F*_0_) contours contribute to word recognition against interfering speech in dyslexic and non-dyslexic children. Thirty-two children with dyslexia and 35 chronological-age-matched control children were tested on the recognition of words in normal sentences versus wordlist sentences with natural versus flat *F*_0_ contours against single-talker interference. The dyslexic children had overall poorer recognition performance than non-dyslexic children. Furthermore, semantic context differentially modulated the effect of *F*_0_ contours on the recognition performances of the two groups. Specifically, compared with flat *F*_0_ contours, natural *F*_0_ contours increased the recognition accuracy of dyslexic children less than non-dyslexic children in the wordlist condition. By contrast, natural *F*_0_ contours increased the recognition accuracy of both groups to a similar extent in the sentence condition. These results indicate that access to semantic context improves the effect of natural *F*_0_ contours on word recognition in adverse listening conditions by dyslexic children who are more impaired in the use of natural *F*_0_ contours during isolated and unrelated word recognition. Our findings have practical implications for communication with dyslexic children when listening conditions are unfavorable.

## Introduction

Although developmental dyslexia is characterized by difficulties in reading and spelling, speech perception deficits have often been reported in dyslexic individuals, especially under adverse or challenging listening conditions. At the phonemic level, a number of studies on categorical perception have showed that phonetic continua were less categorically perceived in dyslexic children than in age-matched controls, suggesting imprecise representations of phoneme categories in dyslexia (Hazan and Barrett, 2000; Blomert et al., 2004; Bogliotti et al., 2008; Cheung et al., 2009; Zhang et al., 2012). At the syllabic level, dyslexic children showed deficits in the identification of naturally produced nonsense disyllables presented in various noisy backgrounds but not in quiet (Ziegler et al., 2009). At the word level, presence of difficulties has also been confirmed in recognition of isolated words in noise by dyslexic adults and children (Brady et al., 1983; Boets et al., 2007; Dole et al., 2012). Taken together, the previous studies indicate that such speech perception difficulties in dyslexia are subtle, which may not be seen in quiet listening conditions partly due to the redundant acoustic cues in speech signals, but tend to emerge in adverse listening conditions with degraded or ambiguous acoustic information.

Previous research has mostly focused on the perception of individual phonemes and isolated words although sentence-level performance would be a better reflection of the linguistic skills for speech communication in natural settings than test results with phonemes and words presented in isolation. Sentence recognition requires extra context-driven semantic, syntactic, and pragmatic processing, which is obviated in the recognition of isolated words. The intelligibility advantage of words in sentences over words in isolation when presented in adverse listening conditions has been confirmed by a number of studies on adults and children without dyslexia (e.g., Dubno et al., 2000; Wang et al., 2013; Jiang et al., 2017). However, only one study has examined sentence-in-noise recognition by dyslexic children to the best of our knowledge. In Nittrouer et al. (2018), dyslexic children aged 7.8 to 12.7 years were required to repeat short sentences consisting of four words presented with speech-shaped noise background. The recognition scores of dyslexic children were lower than those of non-dyslexic children, indicating that speech-in-noise perception in dyslexia is impaired at the sentence level. The sentence materials in their study, however, were specially designed. Specifically, the sentences have correct syntactic structures but lack strong or predictable semantic contextual cues (e.g., Great shelf needs tape). That is, knowledge of morphological and syntactic structures is the main linguistic influence on recognition beyond sensory information. Therefore, it remains unclear whether dyslexic and non-dyslexic children differ in their use of semantic context to aid sentence recognition in adverse listening conditions. In a previous study that adopted phoneme identification task to assess the bias toward real words rather than nonsense words, dyslexic children have been found to rely more on word knowledge to recognize phonemes than non-dyslexic children, indicating that dyslexic children could use some higher-level processing strategies to compensate for their lower-level perceptual difficulties (Reed, 1989). Therefore, one might suspect that the speech perception difficulties in dyslexic children at the level of isolated words may be attenuated when the children are tested with sentences in which semantic context is available.

Speech-in-noise recognition is affected by fundamental frequency (*F*_0_) contours irrespective of whether the target language is a tonal or non-tonal language. Specifically, natural *F*_0_ contours improve intelligibility of speech presented against interference compared with unnaturally inverted or flattened *F*_0_ contours (Laures and Bunton, 2003; Binns and Culling, 2007; Watson and Schlauch, 2008; Miller et al., 2010; Patel et al., 2010). Furthermore, the effect of *F*_0_ contours on speech intelligibility in adverse listening conditions can be modulated by semantic context. In our previous study (Wang et al., 2013), intelligibility of normal sentence and wordlist sentence (a sentence composed of pseudorandomly arranged words but syntactically correct) with natural and flat *F*_0_ contours was examined in adult listeners without dyslexia. The key difference between normal sentence and wordlist sentence is that normal sentence provides sentential semantic context whereas wordlist sentence strips away that context by arranging random lexical items without coherent semantics. The contrastive manipulation of natural and flat *F*_0_ contours is that the former does not make changes to the natural dynamic *F*_0_ contours whereas the latter flattens all *F*_0_ contours, making all the words in the sentence monotonous. Our findings showed that intelligibility increased to a greater extent for normal sentence than wordlist sentence presented at various signal-to-noise ratios (SNRs) when *F*_0_ patterns changed from flat contours to natural contours. Previous studies have revealed that non-tonal language speaking children with dyslexia have impaired abilities to accurately use prosodic properties to disambiguate linguistic structures and to identify the stressed word within an utterance (Marshall et al., 2009). Similarly, Chinese-speaking children with dyslexia are impaired in using *F*_0_ contours to identify lexical tones (Cheung et al., 2009; Zhang et al., 2012). However, whether the detrimental effect of *F*_0_ perception deficit on speech recognition in dyslexia is attenuated by semantic context remains unexplored.

The aim of the present study was to explore the effects of semantic context and *F*_0_ contours on word recognition against interfering speech by comparing Chinese-speaking dyslexic children with chronological age-matched controls. The experiment followed the design of our previous speech intelligibility studies (Jiang et al., 2017; Zhou et al., 2017) by manipulating two factors, namely, semantic context (normal sentence versus wordlist sentence) and *F*_0_ contours (utterances with natural versus flat contours). One possible outcome is that semantic context might be similarly used by dyslexic and non-dyslexic children to aid word recognition regardless of the acoustic manipulation. Another possible outcome is that natural *F*_0_ contours might be differentially used by dyslexic and non-dyslexic children during word recognition due to potential deficits in the dyslexic individuals to use *F*_0_ contours for word segmentation and lexical tone identification. As no previous research has investigated this issue, it remains indeterminate whether semantic context differentially modulates the effect of *F*_0_ contours on word recognition between dyslexic and non-dyslexic children, which constituted the primary motivation for this study.

## Materials and Methods

### Participants

Sixty-seven children were recruited for the study, with 32 showing typical sequelae of developmental dyslexia (18 boys and 14 girls) and 35 being typically developing children (21 boys and 14 girls). All children were normal primary and middle school students in Beijing and were free of neurological, psychiatric, or hearing disorders including attention-deficit/hyperactivity disorder (ADHD) according to parental reports. They all had normal hearing with the threshold of both ears below 25 dB for octave frequencies between 125 and 8000 Hz. The non-dyslexic children were matched with the dyslexics on age and on non-verbal intelligence (according to Raven’s performance IQ). Informed consent was obtained from each of the participants or legal guardians prior to participation. The study was approved by the Institutional Review Board (IRB) of the National Key Laboratory of Cognitive Neuroscience and Learning at Beijing Normal University.

### Diagnosis of Dyslexia

According to the well-established criteria (Shu et al., 2006; Lei et al., 2011; Zhang et al., 2012), three literacy subtests including character recognition, wordlist reading and reading fluency were adopted to identify children with dyslexia. During the character recognition subtest, children were asked to read aloud 150 characters with all learned by Grade 6 primary school children (Shu et al., 2003). During the wordlist reading subtest, children were required to name as quickly as possible 180 words with each of the words composed of two simple and familiar characters. In both subtests, the testing stopped when a child failed 15 items consecutively. Each correctly read character/two-character word was worth one point. During the timed reading fluency subtest that followed the procedure of Landerl et al. (2009), children were asked to read sentences as quickly as possible and judge whether the facts stated in each sentence was correct or not within the time limit of 3 min. There were, in total, 100 sentences with gradually increasing length across the subtest. All the characters in the correctly judged sentences were summed, and each character was worth one point. For inclusion in the dyslexia group, a child would need to have scored one or more standard deviations below the respective age means in all the three subtests. Performance mean scores and standard deviations on the screening subtests are summarized in Table 1 for the two groups.

**TABLE 1:** Demographics, reading, and cognitive measures (±SD) of dyslexic and non-dyslexic children.

	**Dyslexics**	**Controls**	**Statistical test**	***p* value**
*N*	32	35	–	–
Sex (M:F)	18:14	21:14	0.097*^*a*^*	0.8671
Age (years)	12.3 (±1.6)	12.9 (±1.5)	−1.499*^*b*^*	0.1386
Performance IQ	99 (±7)	101 (±9)	−0.939*^*b*^*	0.3512
Character recognition	105 (±18)	134 (± 9)	−8.307*^*b*^*	8.325e−12
Word list reading*^*c*^*	78 (±23)	106 (± 20)	−5.249*^*b*^*	1.857e−6
Reading fluency	265 (±132)	435 (±121)	−5.488*^*b*^*	7.2e−7

*^*a*^Result of chi-square test. ^*b*^Results of two-sample *t*-tests. ^*c*^One dyslexia subject did not complete the wordlist reading test.*

### Word Recognition Test

#### Stimuli

**FIGURE 1:**
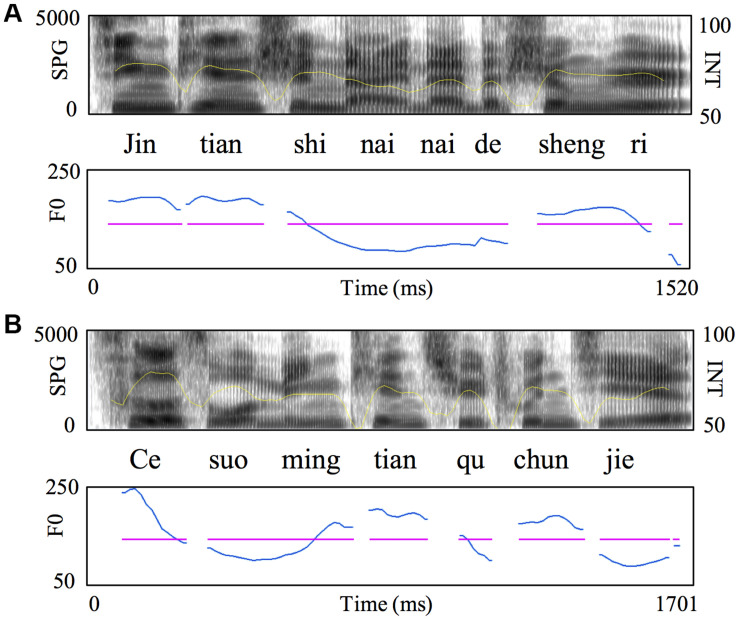
Acoustic properties of example speech stimuli. Broadband spectrograms (SPG, range: 0–5 kHz), intensity envelopes (INT, range: 50–100 dB) and *F*_0_ contours (range: 50–250 Hz; blue: natural; purple, flat) are displayed for **(A)** normal sentence and the pitch-flattened counterpart, and **(B)** word list sentence and the pitch-flattened counterpart. Syllables are marked in *pinyin* (the Chinese phonetic transcription system).

The speech materials were used in our previous studies with native Japanese speakers learning Chinese as a foreign language (Zhang et al., 2016) and Chinese school-age children (Zhou et al., 2017). Cronbach’s alpha values ranged from 0.81 to 0.89 for different types of stimuli in all the studies including the present one, reflecting high internal consistency of each type of stimuli. Specifically, there are four types of stimuli in a 2 × 2 design of variations in semantic context (normal sentence versus wordlist sentence) and *F*_0_ contours (natural *F*_0_ contours versus flat *F*_0_ contours) (Figure 1). The normal sentences consisted of 28 simple declarative Chinese sentences. The wordlist sentences were constructed by pseudorandomly selecting words from the word pool of normal sentences and thus were syntactically correct but lack semantic meanings at the whole sentence level. Normal sentences and wordlist sentences were matched in number of words. Both types of sentences were read by a male native Chinese speaker in a soundproof room with a 44.1-kHz sampling frequency rate and 16-bit digitization. Stimuli with flat *F*_0_ contours were implemented using Praat^1^ to flatten the original *F*_0_ contours at the mean *F*_0_ value of each sentence while preserving other prosodic features such as amplitude envelope and duration. Consonant-misplaced sentences were used as masker sentences to provide energetic masking because they were lexically not meaningful and syntactically incorrect (Xu et al., 2013). The masker sentences were read by a female native Chinese speaker in order to enable the participants to separate easily the target message from the interfering speech. The target and masker sentences were mixed pairwise at the SNR level of +5 dB.

#### Procedures

The experimental protocol followed our previous studies (Wang et al., 2013; Zhou et al., 2017). In a quiet room with ambient noise level no higher than 15 dB sound pressure level, children listened to the stimuli and were required to report orally what they heard. The stimuli were presented through a set of loudspeakers (Edifier R18) with the sound pressure level set at 65 dB calibrated at the listener’s head. Because the sentences with flat *F*_0_ contours were created based on those with natural *F*_0_ contours, we adopted a counterbalanced design to control for possible stimulus order effect. Specifically, each child was presented with half of the total (14/28) stimuli with flat *F*_0_ contours and the other half with natural *F*_0_ contours. Therefore, each child listened to 56 trials in total with 14 trials in each of the four types of stimuli. All the stimuli were presented randomly across listeners with each stimulus heard only once. The task was self-paced and practice was carried out before the actual experiment to familiarize the children with all types of stimuli.

A digital voice recorder was used to record children’s spoken responses. The first author of the present study then scored the responses offline according to a strict score standard. Specifically, the response was considered correct only when each segmental and suprasegmental phoneme of a word was correctly identified by the children. An independent linguist who was blind to the aim of the study checked the scoring and differed in scoring for only five words across all the trials and all the subjects. The two raters reexamined and discussed to resolve the scoring discrepancies for the five words.

## Results

Speech recognition accuracy Table 2 was computed based on a keyword-correct count. Specifically, the number of keywords (i.e., content words) identified correctly by each child was counted and converted to a percentage of the total number of words (Scott et al., 2004; Wang et al., 2013). A 2 × 2 × 2 repeated measures analysis of variance (ANOVA) was conducted with group (dyslexic children versus non-dyslexic controls) as the between-subject factor and semantic context (normal sentence versus wordlist sentence) and *F*_0_ contours (natural *F*_0_ contours versus flat *F*_0_ contours) as the within-subject factors. Results demonstrated that all the three main effects were highly significant: semantic context, *F*(1,65) = 189.567, *p* = 6.12e−21, partial η^2^ = 0.745; *F*_0_ contours, *F*(1,65) = 277.133, *p* = 3.948e−25, partial η^2^ = 0.81; group, *F*(1,65) = 22.554, *p* = 1.167e−5, partial η^2^ = 0.258. These results indicate that both natural *F*_0_ contours and sentential semantic context contribute to better speech recognition by children with and without dyslexia. More importantly, compared with non-dyslexic peers, dyslexic children are impaired in word recognition against interfering speech.

**TABLE 2:** Mean accuracy (±SD) of each condition for the two groups.

	**Controls**		**Dyslexics**	
	**Normal sentence**	**Wordlist sentence**	**Normal sentence**	**Wordlist sentence**
Natural *F*_0_	0.91 (±0.08)	0.74 (±0.16)	0.74 (±0.20)	0.51 (±0.21)
Flat *F*_0_	0.72 (±0.15)	0.50 (±0.19)	0.54 (±0.21)	0.35 (±0.19)

**FIGURE 2:**
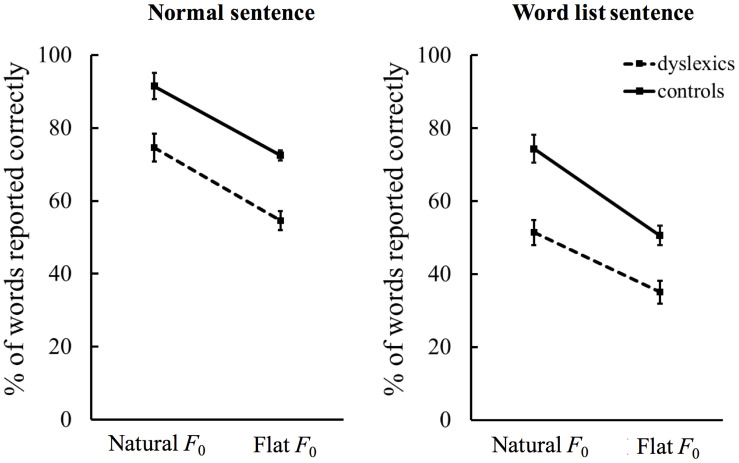
Word-report accuracies of the simple interaction effects carried out on the significant three-way interaction between group, semantic context, and *F*_0_ contours. Error bars represent standard deviation.

ANOVA tests further revealed no significance for all the two-way interaction effects [semantic context × *F*_0_ contours, *F*(1,65) = 0.067, *p* = 0.796, partial η^2^ = 0.0001; semantic context × group, *F*(1,65) = 0.374, *p* = 0.543, partial η^2^ = 0.006; *F*_0_ contours × group, *F*(1,65) = 1.739, *p* = 0.192, partial η^2^ = 0.026]. However, there was a significant three-way interaction effect between group, semantic context, and *F*_0_ contours [*F*(1,65) = 4.586, *p* = 0.036, partial η^2^ = 0.066]. Further analyses, therefore, focused on the significant three-way interaction. Specifically, we decomposed this interaction effect step by step (Maxwell and Delaney, 2004). Firstly, separate two-way ANOVAs were conducted to examine the simple interaction effects. Significant interaction effect between group and *F*_0_ contours was revealed in the wordlist sentence condition [*F*(1,65) = 6.871, *p* = 0.011, partial η^2^ = 0.096], but absent in the normal sentence condition [*F*(1,65) = 0.107, *p* = 0.745, partial η^2^ = 0.002], indicating that semantic context modulates the effect of *F*_0_ contours on word recognition by dyslexic versus non-dyslexic children (Figure 2). Second, follow-up analyses showed that for both wordlist sentence and normal sentence, stimuli with natural *F*_0_ contours were recognized better than those with flat *F*_0_ contours, and moreover, non-dyslexic children performed better than their peers on the recognition of stimuli with natural and flat *F*_0_ contours, consistent with the significant main effects of *F*_0_ contours and group revealed by the original three-way ANOVA.

## Discussion

The current study investigated word recognition against single-talker interfering speech by Chinese-speaking children with dyslexia compared with chronological-age-matched controls. Our results revealed clear evidence for impairment in word recognition in the dyslexic children, and furthermore, semantic context differentially modulated the effect of *F*_0_ contours on word recognition between the two groups.

In the present study, dyslexic children showed much lower performance on the recognition of normal sentence. Specifically, the recognition rates of normal sentence with natural and flat *F*_0_ contours were 74 and 54% in the dyslexic children in contrast to 91 and 72% in the non-dyslexic children. Together with findings of previous studies on the perception of various smaller linguistic units including phonemes, nonsense syllables, and isolated words (Brady et al., 1983; Hazan and Barrett, 2000; Blomert et al., 2004; Boets et al., 2007; Bogliotti et al., 2008; Ziegler et al., 2009; Dole et al., 2012), our results confirmed that in addition to difficulties in reading and spelling, the overall abilities in speech perception is impaired in dyslexia under adverse listening conditions. The beneficial role of semantic context in word recognition by non-dyslexic children has been well established in previous research. For example, children of 4 years old detected mispronounced words in high-predictability sentences better than in low-predictability sentences (Cole and Perfetti, 1980), and children of 7 to 11 years old repeated target words in semantically appropriate contexts more rapidly than in semantically anomalous sentences (Liu et al., 1997). In the present study, the highly significant main effect of semantic context together with the absence of interaction between group and semantic context revealed that dyslexic children used sentential context information to aid word recognition to a similar extent as non-dyslexic children did, thus confirming our prediction.

In tonal languages like Chinese, lexical tones are suprasegmental features, which are phonologically as important as segmental phonemes. As the primary acoustic correlates of Chinese lexical tones, natural *F*_0_ contours play a very important role in word recognition, especially when speech is presented in suboptimal listening conditions or without contextual information (Patel et al., 2010; Wang et al., 2013; Jiang et al., 2017). Our previous study revealed that dyslexic children aged 9–11 years were able to use *F*_0_ contours to identify Chinese lexical tones of isolated syllables presented in quiet although their recognition rates were significantly lower than those of age-matched children without dyslexia (Zhang et al., 2012). In the present study, the highly significant main effect of *F*_0_ contours together with the absence of interaction effect between group and *F*_0_ contours revealed that even in a suboptimal listening condition, dyslexic children showed sensitivity to dynamic *F*_0_ contours to a similar extent as non-dyslexic children did. More importantly, the significant three-way interaction further revealed that semantic context differentially modulated the effect of *F*_0_ contours on word recognition between the two groups. Specifically, in the wordlist condition, dyslexic children showed a smaller recognition advantage of natural *F*_0_ contours over flat *F*_0_ contours compared with non-dyslexic controls, but in the normal sentence condition, both groups showed a similar recognition advantage of natural *F*_0_ contours. Thus, these results extended our previous findings by indicating that semantic context helps dyslexic children compensate to some extent for the perceptual deficit in *F*_0_ contours of isolated syllables and unrelated words. This means that dyslexic children benefited more from semantic context than non-dyslexic children in the use of natural *F*_0_ contours to assist word recognition, reflecting the interactive contributions of top-down semantic processing and bottom-up phonetic processing to word recognition by dyslexic children at the SNR level (i.e., +5 dB) adopted in the present study.

The current results are limited in several aspects. Firstly, no linguistic subskills (e.g., phonological awareness and vocabulary) or cognitive competences (e.g., short-term memory and working memory) were measured. Therefore, it is unclear whether the overall difficulties in word recognition against single-talker interference simply reflects one component of the broadly based language deficits in dyslexia or the deficits in speech perception and visual forms of language arise from a single factor (e.g., auditory dysfunction versus phonological awareness as suggested by many previous studies on dyslexia) (Tallal, 1980; Snowling, 2000; Ramus et al., 2003; Vellutino et al., 2004; Nittrouer et al., 2011). Our results also did not address how various interrelated linguistic and cognitive skills benefit, separately or interactively with semantic context, the use of natural *F*_0_ contours during word recognition by dyslexic children. Secondly, the interfering speech used in the present study are consonant-misplaced sentences that are syntactically incorrect and semantically anomalous at the sentence level. One concern might arise with the masking effects of this specific type of stimuli on the current results. Different kinds of maskers (e.g., white noise, speech-shaped noise, one- and multi-talker babbles and time-reversed speech) have different effects on speech recognition because they differ to a great extent in distracting listeners and masking targets (Garcia Lecumberri and Cooke, 2006; Dole et al., 2012). Furthermore, male and female voices were designated to be the target and masker stimuli, respectively, to make the instructions easy to follow for our participants. Further research using different kinds of interfering sounds at various SNRs and speech of the same and different genders as target and masker are necessary to clarify how the effects of semantic and *F*_0_ contours on word recognition by dyslexic children is affected by characteristics of targets and maskers. Thirdly, reading experience may likely be reduced in dyslexics relative to controls, which would conceivably contribute to the experimental outcomes. A further complication is that the current findings might be affected to some extent by comorbidity of dyslexia and ADHD in some of the children as we only relied on parental reports without administering a strict diagnostic test to exclude children with ADHD. Previous research has indicated that comorbidity of dyslexia and ADHD can lead to deficits in language processes such as phonological awareness, listening comprehension, and verbal working-memory (Tannock et al., 2000; McInnes et al., 2003; Tiffin-Richards et al., 2008). How semantic context and natural *F*_0_ contours may affect word recognition differently in dyslexic children with and without ADHD warrants further investigation. Finally, caution is needed in interpreting the significant three-way interaction effect due to the small effect size. Future studies with a larger sample size and more specific inclusion and exclusion criteria are needed to verify the different effects of semantic context and *F*_0_ contours on word recognition in children with dyslexia.

## Conclusion

The current results demonstrate the important roles of semantic context and natural *F*_0_ contours in word recognition by dyslexic children. Our findings imply that one effective means of enhancing speech intelligibility under adverse listening conditions during communication with dyslexic children is to introduce modification of the talker’s speech. Specifically, intelligibility of speech directed at dyslexic children may be enhanced through changes in the speaking style with an emphasis on the use of dynamic *F*_0_ contours and the sentential semantic context with immediate beneficial effects.

## Data Availability Statement

The original contributions presented in the study are included in the article/supplementary material, further inquiries can be directed to the corresponding author.

## Ethics Statement

The studies involving human participants were reviewed and approved by Institutional Review Board (IRB) of the National Key Laboratory of Cognitive Neuroscience and Learning at Beijing Normal University. Written informed consent to participate in this study was provided by the participants’ legal guardian/next of kin.

## Author Contributions

LZ, YL, and HZ: conceptualization, data collection, software, analysis, and manuscript preparation. HS and YZ: conceptualization and manuscript preparation. All authors contributed to the article and approved the submitted version.

## Conflict of Interest

The authors declare that the research was conducted in the absence of any commercial or financial relationships that could be construed as a potential conflict of interest.

## Footnotes

1 https://www.fon.hum.uva.nl/praat/
